# The Effectiveness of Parent Training as a Treatment for Preschool Attention-Deficit/Hyperactivity Disorder: Study Protocol for a Randomized Controlled, Multicenter Trial of the New Forest Parenting Program in Everyday Clinical Practice

**DOI:** 10.2196/resprot.5319

**Published:** 2016-04-13

**Authors:** Anne-Mette Lange, David Daley, Morten Frydenberg, Charlotte U Rask, Edmund Sonuga-Barke, Per H Thomsen

**Affiliations:** ^1^ Centre for Child & Adolescent Psychiatry Research Department Aarhus University Hospital Risskov Denmark; ^2^ Division of Psychiatry and Applied Psychology School of Medicine & Centre for ADHD and Neurodevelopmental Disorders Across the Lifespan & NIHR MindTech Health Care Technology Cooperative, Institute of Mental Health University of Nottingham Nottingham United Kingdom; ^3^ Section for Biostatistics Department of Public Health Aarhus University Aarhus Denmark; ^4^ Research Clinic for Functional Disorders and Psychosomatics Aarhus University Hospital Aarhus Denmark; ^5^ Developmental Brain-Behaviour Laboratory Academic Unit of Psychology University of Southampton Southampton United Kingdom; ^6^ Research Department Centre for Child & Adolescent Psychiatry Aarhus University Hospital Risskov Denmark; ^7^ Department of Experimental Clinical & Health Psychology, Ghent University Ghent Belgium; ^8^ Faculty of Medicine Trondheim University Trondheim Norway

**Keywords:** ADHD, preschool, child, treatment, parents, parent training, psycho-social, RCT, clinical, psychological, multi-centre, TAU, non-pharmacological

## Abstract

**Background:**

Parent training is recommended as the first-line treatment for attention-deficit/hyperactivity disorder (ADHD) in preschool children. The New Forest Parenting Programme (NFPP) is an evidence-based parenting program developed specifically to target preschool ADHD.

**Objective:**

The objective of this trial is to investigate whether the NFPP can be effectively delivered for children referred through official community pathways in everyday clinical practice.

**Methods:**

A multicenter randomized controlled parallel arm trial design is employed. There are two treatment arms, NFPP and treatment as usual. NFPP consists of eight individually delivered parenting sessions, where the child attends during three of the sessions. Outcomes are examined at three time points (T1, T2, T3): T1 (baseline), T2 (week 12, post intervention), and T3 (6 month follow/up). 140 children between the ages of 3-7, with a clinical diagnosis of ADHD, informed by the Development and Well Being Assessment, and recruited from three child and adolescent psychiatry departments in Denmark will take part. Randomization is on a 1:1 basis, stratified for age and gender.

**Results:**

The primary endpoint is change in ADHD symptoms as measured by the Preschool ADHD-Rating Scale (ADHD-RS) by T2. Secondary outcome measures include: effects on this measure at T3 and T2 and T3 measures of teacher reported Preschool ADHD-RS scores, parent and teacher rated scores on the Strength & Difficulties Questionnaire, direct observation of ADHD behaviors during Child’s Solo Play, observation of parent-child interaction, parent sense of competence, and family stress. Results will be reported using the standards set out in the Consolidated Standards of Reporting Trials Statement for Randomized Controlled Trials of nonpharmacological treatments.

**Conclusions:**

The trial will provide evidence as to whether NFPP is a more effective treatment for preschool ADHD than the treatment usually offered in everyday clinical practice.

**Trial Registration:**

ClinicalTrials.gov NCT01684644; https://clinicaltrials.gov/ct2/show/NCT01684644?term= NCT01684644&rank=1 (Archived by WebCite at http://www.webcitation/6eOOAe8Qe)

## Introduction

### Attention Deficit/Hyperactivity-Disorder Costs for Children

Attention deficit/hyperactivity-disorder (ADHD) is a neuro-developmental disorder with symptoms frequently occurring in early childhood, however, there is a parenting program that was developed specifically for the management of ADHD in preschool children, noted below [1,2]. ADHD is one of the most common psychiatric disorders in childhood [3] and the most common reason for referral to child mental health services (CAMHS) [4]. Studies indicate that ADHD is as common in preschoolers as it is in school-age children, with population based prevalence rates ranging between 2 and 5% [5,6]. Impairment is equally common [7], including marked impairments in relationships with parents, siblings, and peers [8-11]; social and preacademic skills [12]; and neuropsychological functioning [13]. Preschool children with ADHD are at greater risk of placement in special educational classes and use more special needs services [14]. Young children with ADHD are more likely to suffer physical injury and accidental poisoning related to impulsive behaviors [7]. ADHD symptoms in preschoolers show persistence over time [15,16]. Lahey et al [17] found that 4-6 year old children who met full criteria for ADHD were highly likely to continue to meet criteria 3 years later. Preschool hyperactivity is associated with long-term economic burden. Evidence from a longitudinal health economic analysis of costs incurred across childhood, adolescence, and young adulthood found significantly higher costs for this group of individuals in areas of mental health, education, social service, and the criminal justice system [18]. In a large register-based study, it was found that childhood ADHD, by the time the child is 10, reduces parental socioeconomic status by lowering labor supply and earnings, and reduces parental relationship stability [19]. Left untreated, preschool ADHD is associated with long-term poor outcomes for the patient [20]. Hence, it is of central public priority generally, and for CAMHS specifically, to offer evidence-based and cost-effective treatments at an early stage of development in an attempt to reduce the long-term risks and change the negative trajectories that ADHD presents to the individual and to society [2].

### Behavior-Based Treatment

Although medication is recommended as the front-line treatment for older children and adolescents with ADHD, international treatment guidelines do not recommend pharmacological therapies for preschool ADHD [21,22]. Indeed, available data suggest that stimulant medication such as methylphenidate is less efficacious in the preschool years and side effects are more common [23]. Long-term effects on growth and brain development are currently not definitively established. Because of these factors, psychosocial behavioral treatments for ADHD, including behavioral parent training and behaviorally based day-care interventions, are recommended as first-line treatments for ADHD in the preschool years [21]. A number of psychosocial interventions have been applied and evaluated for the treatment of preschool ADHD. A recent systematic review found that behavioral parent training is valuable as a treatment for preschool ADHD [24]. Furthermore, Sonuga-Barke et al [25] found that trials evaluating behaviorally based parent interventions had the largest effects for preschool children with ADHD.

Whereas many behaviorally based interventions are generic in nature, in that they were originally developed to help parents manage preschoolers’ oppositional and challenging behavior, the New Forest Parenting Programme (NFPP) [1] is a parenting program that was developed specifically for the management of ADHD in preschool children. In addition to behavioral strategies, it includes ideas for games and activities aimed at targeting some of the self-regulatory and attention deficits that cause impairment in the condition. Supporting the child’s development through parental scaffolding is a key component of the NFPP, and everyday play scenarios in the home constitute opportunities for parents to train and improve the child’s ADHD symptoms. A single practitioner delivers NFPP to families in their home during eight weekly sessions.

Each session lasts approximately 90 minutes and is either for the parent only (five sessions) or the parent and child together. In an evaluation of the NFPP, Sonuga-Barke et al [1] found that the intervention reduced ADHD symptoms as reported by parents and improved behavior during a play observation task, as rated by blinded observers. Similarly, Thompson et al [26] reported that NFPP improved parent-reported ADHD symptoms in a sample of preschool children with ADHD.

At present, the evidence of the effect of NFPP is based on efficacy research carried out by the expert team of researchers and clinicians who developed the program originally, with therapists receiving support from lead investigators, and with children who have not been referred for treatment through regular community pathways. Yet, it is unexplored if the findings from the original research can be replicated in different clinical and cultural contexts, including in a different language. This leaves open the question of whether effects shown in specialist research led services can be translated in the everyday practice of established clinical CAMHS [27]. In a review of implementation studies [28], it was shown that there is a risk that interventions will lose impact and potency when implemented in everyday clinical settings. Hence, it is emphasised that the transition of efficacious treatments has to be carefully planned and adapted to the clinical setting in which the treatment is being implemented [27,29].

Evidence-based parent training interventions specifically designed for preschool ADHD, such as the NFPP, are not currently provided by Danish CAMHS. The development and implementation of effective psychosocial treatments for young children with ADHD represent an important health policy objective. The trial described in this protocol is designed to evaluate the effectiveness of the NFPP in the treatment of young children diagnosed with ADHD referred through established clinical pathways to child psychiatry departments in Denmark. It is the first time that the NFPP has been tested in a European country outside the United Kingdom, and the first time the NFPP has been tested on children referred through established clinical pathways to tertiary child psychiatry services, to our knowledge. For these purposes, the NFPP program has been adjusted to fit the clinical and cultural context of Danish child and adolescent psychiatry services.

### Aim of the Study

The objective of the trial is to examine whether the NFPP can be implemented effectively as a treatment for preschool ADHD in everyday clinical settings of existing services. To do this, we will examine ADHD and other outcomes at the end of treatment and also at a 6-month follow-up.

## Methods

### Trial Design

A randomized controlled trial will be carried out to investigate the effectiveness of the NFPP against a treatment as usual condition (TAU) in the treatment of ADHD in a clinical sample of young children referred to, assessed, and diagnosed at tertiary, preschool child psychiatry services in Denmark. The effectiveness of the NFPP intervention will be examined at three time points: T1 (baseline), T2 (week 12, post intervention), and T3 (6 month follow/up).

### Ethics

The study is approved by the Ethics Committee for Central Danish Region (No: 1-10-72-140-12), and is approved by the Danish Data Protection Agency. The trial is registered at ClinicalTrial.gov identity no: NCT01684644, September 4, 2012.

### Setting

Participants will be enlisted from the regional, hospital-based child psychiatry departments for preschool children at the three participating centers in the trial: Centre for Child and Adolescent Psychiatry-Risskov; Centre for Child and Adolescent Psychiatry-Herning; and Centre for Child and Adolescent Psychiatry-Glostrup. The departments are highly specialized, tertiary CAMH services, and lie at the end of the referral pathway for young children with mental health problems in Denmark. Prior to their referral to specialist child psychiatry services, children will usually have received an initial assessment and intervention by primary care professionals, such as an early years educational psychologist, a community pediatrician, and social services.

### Eligibility Criteria

#### Inclusion Criteria

Inclusion criteria are: (1) child age between 3-7 years old; (2) child must have received a clinical diagnosis of ADHD, as informed by the Development and Well-Being Assessment (DAWBA) [4]; and (3) Danish must be first language spoken in the home.

#### Exclusion Criteria

Child exclusion criteria are: (1) intellectual disabilities (IQ < 70); (2) diagnosis of autism spectrum disorders; and (3) child currently receiving pharmacological or other psychosocial treatment for ADHD.

Parent exclusion criteria are: (1) severe psychiatric disorder (eg, untreated psychosis or untreated bipolar or severe depressive disorder) and (2) severe social adversity in the home, as defined by active child protection issues.

### Recruitment Procedures

A total of 140 children (age 3-7) referred through official referral routes to a general child psychiatry service for preschool children and their parents will be recruited into the trial. A clinical psychologist or a specialist in child and adolescent psychiatry initially screens all referrals for eligibility. Referrals indicating noneligibility, due to direct descriptions of exclusion criteria in the referral letter, receive standard clinical assessment from the preschool child psychiatry team. At this point, all other referrals are regarded as eligible and parents are sent a letter including a personal access code asking them to complete the Internet DAWBA [4] or the Preschool DAWBA (DAWBA-preschool for 2-4 year olds) [30]. The DAWBA generates computerized diagnostic profiles, which are scored by child and adolescent psychiatrists and clinical psychologists trained in DAWBA rating. The DAWBA is employed at all three participating sites in an attempt to homogenize diagnostic practices and is used to compliment and inform the clinical, diagnostic assessment process.

In conjunction with the DAWBA, eligible children will undergo standard clinical assessment at the referral site. For referrals to preschool child psychiatry services in Denmark, this standard clinical assessment usually consists of: interview with parents regarding the child’s developmental history and current difficulties; child cognitive assessment and neuropsychological tests or neuropsychological tests; child medical examination; and, at Risskov and Herning, a semistructured observation of the child at the child’s day-care facility. Results from clinical assessments and results from the Internet DAWBA are presented and discussed at weekly multidisciplinary clinical team meetings where a specialist doctor in child and adolescent psychiatry issues final clinical diagnoses.

**Figure 1:**
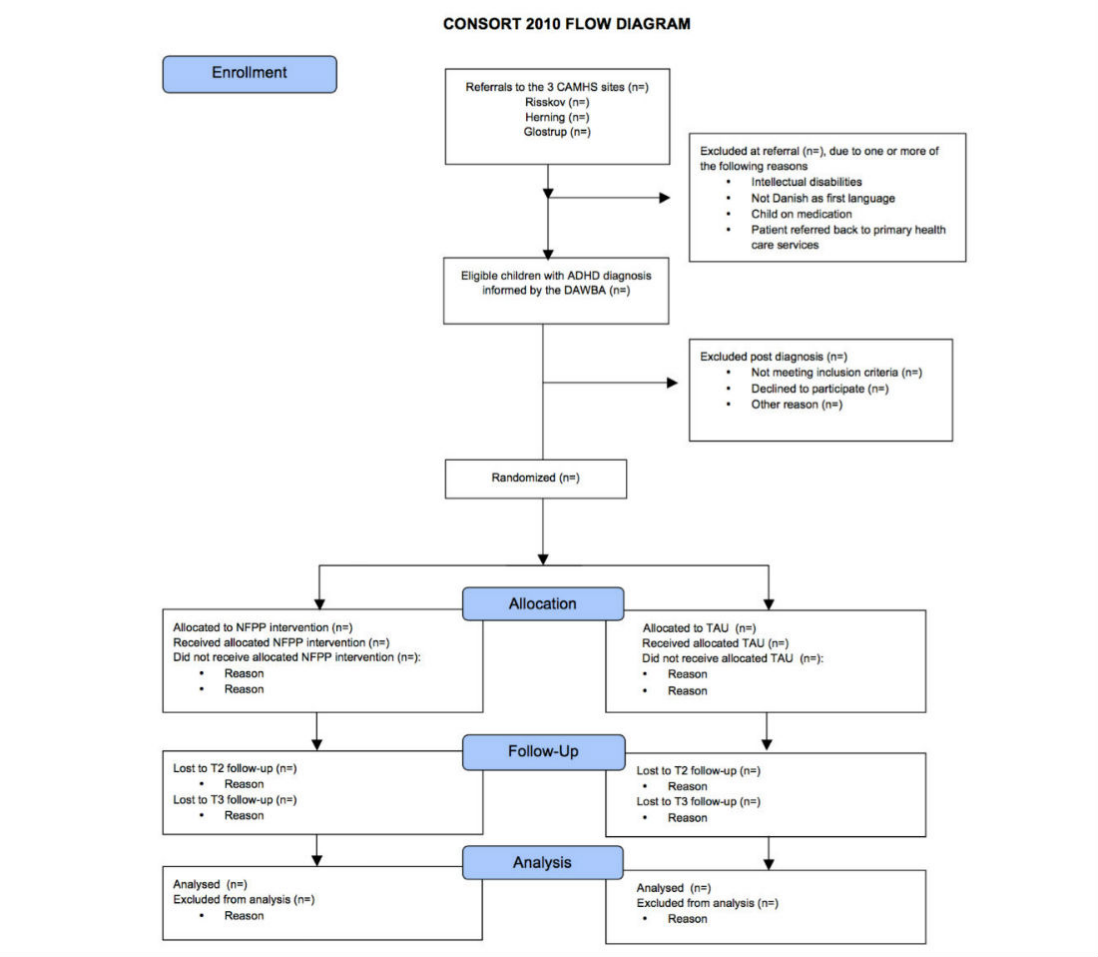
Consolidated Standards of Reporting Trials (CONSORT) flow diagram of recruitment. Child mental health services (CAMHS); attention deficit/hyperactivity-disorder (ADHD); Development and Well-Being Assessment (DAWBA); New Forest Parenting Programme (NFPP); treatment as usual (TAU); T2 (week 12, post intervention); and T3 (6 month follow/up).

Parents of children meeting the research criteria are invited to join the study, and are included in the trial, once written consent is obtained. Figure 1 shows the recruitment flow according to Consolidated Standards of Reporting Trials (CONSORT) guidelines [31].

### Randomization Procedures

Since outcome may be dependent on treatment center and regional variations in referral pathways, as well as gender and age of the child, the study uses stratified allocation to treatment condition to ensure a balanced number of participants over conditions. Randomization is based on the defined strata as well as on the assigned treatment condition of earlier randomized participants within the strata. Parents will be randomized to one of the two arms using an allocation ratio of 1:1 for NFPP and TAU. Randomization to NFPP and TAU (1:1) will be generated by a Web-based randomization computer program within the Internet data management service Trialpartner [32], which allows for on-the-spot randomization of participants into an arm of the study. Randomization is done in blocks of size four or six and in 12 strata defined by center, gender, and age of the child (3-5 and 6-7 years). Once the parent has provided written informed consent to participate in the study, the administrator completes participant registration on the Internet in Trialpartner and enters a randomization request, at which point the randomization program randomly assigns the treatment condition, for example, NFPP or TAU, to the respective participant ID number. Trialpartner simultaneously generates an automated email message for the administrator and the main investigator of the study, in which participant ID, site, and treatment allocation is outlined. Parents are informed of their respective treatment condition following completion of the T1 visit, so that the first assessment is not influenced by participant’s knowledge of treatment condition. Information about treatment allocation is delivered to parents in a telephone call by the researcher who initially informed the parent of the study. 

### Data Management

All T1, T2, and T3 assessment schedules administered to parents and to day-care staff are set up electronically in Trialpartner [32] by the The Data Management Unit in The Central Denmark Region and are administered for completion on the Internet to parents and day-care staff by a research assistant. All data are stored and managed by The Data Management Unit in The Central Denmark Region. Data will remain locked until all participants have completed T3.

### Blinding

Research assistants carrying out research assessments and observations at the three time-points will be blinded. Parents are instructed not to reveal treatment allocation to research assistants. All raters of videotaped observations will be blinded. Research assistants and raters are postgraduate psychology students and newly qualified psychologists. Parents, NFPP therapists, NFPP supervisor, and TAU mental health professionals will not be blinded. Efforts will be made to keep teachers blind to treatment allocation. Yet full blinding cannot be guaranteed, as participants may spontaneously talk about treatment to teachers.

### Sample Size Estimation

The primary endpoint is parent ADHD ratings at T2. Sample size has been estimated at 126 children to obtain power at 0.80, ES=.50 (Cohen’s d). The usual convention that is generally proposed and accepted for randomized trials is that power in a study ought to be 0.80 when ES is estimated at 0.50 [33]. An estimated effect size of 0.5 is deemed reasonable for a trial evaluating the effect of parenting interventions in the treatment of ADHD. By comparison, a recent meta-analysis of parent training interventions for ADHD obtained an ES of 0.4 [34].

For a design comparing two groups, for example, NFPP versus TAU, and allowing for a participant drop out of 10%, a total of 140 children, for example, a sample of 70 children per group is needed to attain a power of 0.80 [35]. A drop out rate of 7% was reported in a Dutch parent training trial implemented in routine clinical CAMHS with a similar age group [36]. In this light, the present trial has chosen to operate with a potential drop out rate of 10%. The preschool child psychiatry sites at Risskov and Herning are expected to enroll 2/3 of participants and the Glostrup site is expected to enroll 1/3 of participants for the trial.

### Treatment Conditions

#### Adapted New Forest Parenting Programme

The original NFPP [1] was developed to be delivered in the child’s home over the course of 8 sessions. However, the catchment areas of the three Danish hospital-based sites participating in this study cover large geographical areas. The distance between the furthermost points in the catchment area of Risskov, for example, is in excess of 150 km, which makes home-visits very time consuming and difficult to carry out to the degree outlined in the original NFPP protocol. Second, child psychiatric services in Denmark do not have a tradition of offering home-based assessment or treatment. As a rule, these are offered on an outpatient basis. To optimize the acceptability and deliverability of the NFPP in Danish CAMHS, the current study has made changes to the delivery of the original program and offers the majority of the 8 treatment sessions in the clinic. The third and the fifth treatment sessions, where the child is present, will be offered in the home. The other six sessions will be offered in the clinic in designated child and family friendly treatment rooms. The content of the NFPP manual remains otherwise unchanged.

The NFPP is centered around five broad themes [37].

1. The importance of psychoeducation about the nature and manifestation of preschool ADHD in order for parents to understand reasons for the child’s behavior.
2. Emphasis on scaffolding to help parents work from the child’s level of development and skills.
3. The importance of parent-child interaction, and how it might be enhanced to support child development and reduce parental stress.
4. Guiding parents in the use of behavioral strategies to improve behavior and ADHD symptoms.
5. Training to improve child’s ADHD related, neuropsychological deficits, where parents are instructed to play with the child using attention training games, and helping the child manage delay, waiting, and self-regulation.

The NFPP manual, course materials, hand-outs, and a treatment DVD have been translated from English into Danish by a professional translation agency and A-ML with permission from the original developers [26]. The original developers have approved all corrections or changes to original wording.

In this trial, families are also given basic play materials that are recommended in the manual for use in specific training tasks with the child, for example, two card games and a special relaxation mat for the child.

#### Treatment as Usual

TAU is likely to be different at the three sites, reflecting the different clinical practice and traditions in everyday outpatient child psychiatry in Denmark. In Risskov, TAU traditionally consists of three sessions of psychoeducation delivered in groups of parents to referred children diagnosed with ADHD. In Herning, TAU typically consists of one individual session with parents, following diagnosis, and one psychoeducation session in a group of parents. In Glostrup, it consists of four psychoeducation sessions in groups for parents. Children do not participate in the psychoeducation groups at any of these services. Clinical decisions may be taken in individual cases that give rise to intrasite variations in TAU. Based on the individual clinical need of the child and the parents, treatment may occasionally be offered by a mental health professional through 2-3 individual sessions to parents. TAU at all sites is carried out by existing clinic staff with many years of clinical experience in relation to preschool ADHD. The nature of treatment provided to parents in the TAU groups will be carefully recorded.

#### Fidelity to Treatment Manual

The NFPP intervention is manual based. All treatment sessions will be videotaped. To verify treatment fidelity based on the NFPP intervention manual, independent raters will code a number of treatment sessions per therapist.

#### New Forest Parenting Programme Training of Danish Therapists and Supervisor

During 2010 and 2011, the lead investigator A-ML participated during two NFPP training events held by the developers of the NFPP, for example, Dr Margaret Thompson (MT) and Ms Cathy Laver-Bradbury’s (CLB), University of Southampton in the United Kingdom. A-ML subsequently saw two young children diagnosed with ADHD and their parents from the preschool child psychiatry clinic in Risskov for 8 sessions NFPP under close supervision from MT and CLB. All treatment sessions were videotaped and translated in order for MT and CLB to supervise A-ML to achieve NFPP therapist certification. Following therapist certification, A-ML embarked upon NFPP supervisor certification and received biweekly supervision from MT and CLB in preparation to supervise Danish therapists employed in this trial.

A total of four therapists: a clinical psychologist and a pedagog from the preschool team at the Glostrup site and a clinical psychologist and a nurse specialist from the Risskov site are employed to train as NFPP therapists for the trial. They have all undergone a 4-day NFPP training course conducted by MT and CLB. Following training, all four therapists provided NFPP treatment to two families each, and received weekly supervision in relation to these families by A-ML. MT and CLB conducted a one-day top up training session after six months, in order to clarify specific questions in relation to the NFPP intervention. Therapists will receive 2-hour clinical supervision sessions in groups of two by A-ML throughout the trial. In turn, A-ML will receive biweekly supervisor supervision via Skype calls by MT and CLB to ensure adherence to the NFPP.

### Assessment and Outcome Measures

Measures administered in this trial are listed in Table 1.

**Table 1:** Trial outcome measures.

Measures				Collected
**Primary outcome measure**				
	**ADHD symptoms**			
			Preschool ADHD-RS (parent rated) [38]	T1, T2, T3
**Secondary outcome measures**				
	**ADHD symptoms**			
			Preschool ADHD-RS (teacher rated) [38]	T1, T2, T3
			Child Solo Play - observation measure	T1, T2, T3
			Behavioral symptoms and impact	
			SDQ^a^P2-4 & P4-16 - SDQ^a^and impact supplement (parent rated) [30,39]	T1, T2, T3
			SDQ^a^T2-4 & T4-16 - SDQ^a^and impact supplement (teacher rated) [30,39]	T1, T2, T3
		**Parent ADHD**		
			The Adult ADHD Self-Report Scale (ASRS-V1.1) [40]	T1
		**Perceived parenting**		
			PSOC^b^[41]	T1, T2, T3
			FSI^c^[42]	T1, T2, T3
		**Positive and constructive parenting**		
			GIPCI^e^(jigsaw/tidy up/freeplay) observation measure [43]	T1, T2, T3
		**Parent mental health**		
			GHQ^d^:12 [44]	T1, T2, T3

^a^ SDQ: Strength and Difficulties Questionnaire

^b^PSOC: Parenting Sense of Competence Scale

^c^FSI: Family Strain Index

^d^ GHQ: General Health Questionnaire

^e^ GIPCI: Global Impressions of Parent-Child Interactions

### Primary Outcome Measure

#### Child Attention Deficit / Hyperactivity-Disorder: Attention Deficit / Hyperactivity-Disorder Rating Scale-IV–Preschool Version

The ADHD Rating Scale (ADHD RS)-IV-Preschool Version [38] is a questionnaire adapted for the use in preschoolers from the 18-item ADHD-RS-IV [45]. In this preschool version, symptom statements have been modified to be more appropriate to the developmental level of preschoolers. The scale includes 18 behavioral descriptors of ADHD as determined by the Diagnostic and Statistical Manual of Mental Disorders (DSM-IV) [46]. Raters can consist of parents and teachers who respond to each question on a 4-point scale from 0 (not at all) to 3 (very often) to reflect the child’s behavior. Scores can be obtained of Inattention, Hyperactive/Impulsive, and Total Scales. Internal consistency for the three scales has been established in the range from .92 to .95 on the Teacher Version and from .85 to .92 on the Parent Version. Test-retest reliability correlations for the three scales ranged from .93 to .96 for the Teacher Version and between .8 and .87 for the Parent Version [38].

#### Attention Deficit / Hyperactivity-Disorder Rating Scale-IV-Preschool Version Translated

The ADHD RS-IV-Preschool Version has been translated into Danish for the purpose of the present study with permission from and formal approval by Kara McGoey (see Multimedia Appendix 1) according to international guidelines for the translation of questionnaires [47].

### Secondary Outcome Measures

#### Child Solo Play

The Child Solo Play [1] instrument is an independent, direct observation measure administered by a research assistant during 5 minutes of solo play with a standard activity, multipurpose toy. Patterns of attending to and switching from one activity to another during play with the toy are measured and indexed in terms of attention and engagement. High index scores represent more attention and less switching. Good test-retest reliability (Pearson r=.81), interrater reliability (Pearson r=.76), and validity—discriminating children with ADHD from non-ADHD children—have been established. Research assistants, for example, postgraduate psychology students and newly qualified psychologists, employed in the trial are trained in the administration and scoring of Child’s Solo Play by the original developer of the measure and coauthor on this paper, DD.

***Parenting Sense of Competence Scale*** The Parenting Sense of Competence Scale (PSOC) [41] is the most frequently used questionnaire applied to measure parental self-efficacy and satisfaction [48]. It measures parental competence on two dimensions: Satisfaction and Efficacy. Separate scales are developed for mothers and fathers, who respond to 16 questions on a 6-point Likert-scale (ranging from strongly agree to strongly disagree) [1,6]. There are 9 questions that are related to Satisfaction and seven are related to Efficacy. The Satisfaction section examines parents’ anxiety, motivation, and frustration, while the Efficacy section looks at parents’ competence, capability levels, and problem-solving abilities in their parental role. A number of studies have demonstrated the scale’s good psychometric properties reporting internal consistency of .75 [41], and internal consistencies of .72 (mothers) and .76 (fathers) [48]. Charlotte Johnston gave permission and approval for the PSOC to be translated for the purpose of the present study (see Multimedia Appendix 4) according to international guidelines for the translation of questionnaires [47].

#### Strength and Difficulties Questionnaire Parent and Teacher Version

The Strength and Difficulties Questionnaire (SDQ) [39] is a brief behavioral five factor instrument developed to assess emotional and behavioral problems in children. The SDQ consists of 25 questions scored on a 3 point Likert scale (“not true”, “somewhat true”, “certainly true”). The questions cover five domains of child psychopathology by five subscales: hyperactivity/inattention, conduct problems, emotional symptoms, peer relationship problems, and prosocial behavior. Each area is covered by five questions. In addition, the SDQ contains an impact supplement inquiring about distress, burden, and impairment of the child. The questionnaire is widely used for clinical as well as research [49]. The standard SDQ is developed for use in the age-range of 4-17 years. The early years SDQ is developed for use in the age range of 2-4 years. A teacher version is developed for each scale, and a scale for completion by the young person is developed for use in the age range 11-17.

The SDQ has been used extensively in European as well as non-European contexts, and has been translated into more than 60 languages [50]. A recent review of the psychometric properties of the parent and teacher versions of the SDQ included 48 studies from 17 different cultural settings and a total of 131,223 raters [50]. The internal reliability and factor structure of the Danish versions of the SDQ were established in a sample of 71,840 parent and teacher raters of 5-, 7-, and 10- to 12-year-old children included in four large-scale Danish cohorts [51]. The predictive validity of the SDQ has been demonstrated in another large Danish study where the SDQ was shown to identify a group of children with highly increased risk of later being diagnosed and treated for ADHD or treated for ADHD in school age [52].

The early years SDQ—parent and teacher version Goodman [30]—has been translated into Danish for the purpose of the present study with personal permission and approval by Robert Goodman (see Multimedia Appendix 2) following standard guidelines for the translation of questionnaires [47]. The early years SDQ has shown good psychometric properties in a recent study [53].

***Family Strain Index*** The Family Strain Index (FSI) [42] is a 6-item parent-report questionnaire developed to assess the effects of ADHD on families to better understand and address the level of stress, strain, and burden that families experience. The FSI is designed to measure two major aspects of stress and demand, for example, the “emotional” and the “restrictiveness” experiences in the context of living with a child with ADHD. The FSI has demonstrated good internal consistency across the six items (Cronbach alpha= .83 to .87) and for the full scale (Cronbach alpha=.87) [42].

The FSI has been translated into Danish for the purpose of the present study (see Multimedia Appendix 3) following standard guidelines for the translation of questionnaires [47] with permission from and approval by the original developer Anne Riley.

#### Global Impressions of Parent-Child Interactions

The Global Impressions of Parent-Child Interactions (GIPCI-R) [43] is a direct, semistructured observation schedule developed to evaluate parent and child interaction. Parent-Child dyads are videotaped in 15 minutes sessions during three tasks, each of 5 minutes duration: “jigsaw”, “free play”, and “tidy-up”. Videotapes are rated using the GIPCI coding manual at a later stage by a rater who is blinded to the randomization of the child. Child behavior items include: respect, disruptive, social skills, and destruction. Parents items rated include: responsiveness, warmth, praise, enjoyment, scaffolding, criticism, and punishment. Ratings produce global summary scores for child and parent with a higher score reflecting a more positive outcome. Thompson et al [26] found adequate interrater reliability for child scores (.62: range .48-.77) and for parent scores (.64: range .48 to .79). Good internal consistency was established for parent and child scales (.84; .87). Training in the administration and scoring is carried out by one of the authors of this paper, DD.

#### The Adult Attention Deficit/Hyperactivity-Disorder Self-Report Scale

The Adult ADHD Self-Report Scale (ADRS-v1.1) is an 18-item Symptom Checklist covering the 18 DSM-IV-TR criteria for ADHD. The scale has been developed in conjunction with the World Health Organization (WHO), and the Workgroup on Adult ADHD [40]. The first 6 questions have been found most sensitive in the screening for ADHD in adults [54]. The 18-item scale has been translated into Danish and has been approved by the original investigators of the WHO Work Group. The scale provides a method of identifying ADHD symptoms in adults and is a powerful tool to discriminate DSM-IV cases from noncases [54]. The scale has good psychometric properties and is widely used in scientific research [55]. The validity and clinical feasibility of the Danish version of the ADHD-RS has been demonstrated in a multicenter study [56]. The measure will be used to evaluate the extent to which parental ADHD symptoms are associated to outcome [57].

#### The General Health Questionnaire

The General Health Questionnaire (GHQ) [44] is the most common assessment measure for assessing of mental well-being in adults. Developed as a screening tool to detect those likely to have or be at risk of developing psychiatric disorders, it is a measure of the common mental health problems/domains of depression, anxiety, somatic symptoms, and social withdrawal. It is available in a variety of versions using 12, 28, 30, or 60 items, which have all been translated into Danish. The present study will use the 12-item version GHQ:12. The measure will be used to evaluate the extent to which parental mental health is associated to outcome [58].

## Results

### Statistical Analysis

The data will be analyzed on an intention to treat basis. All outcomes will be analyzed with a repeated measure model with a randomization arm, gender, age (above or below 5 year), center, year, and a random level for each child as covariates. We will estimate the effect of the intervention at T2 (intervention, TAU) in terms of the difference between the change from T1-T2 and its effect at T3 in terms of change between the T3-T1. Missing data will be imputed assuming Missingness At Random in relevant analysis models including additional control variables if relevant. Outcomes with more than fifty percent missing will not be analyzed. To examine the effect of outliers, a sensitivity analysis will be performed excluding observation with residuals exceeding 2.5*SD. In addition, four other sensitivity analyses will be performed for each analysis exploring how sensitive the results are for reasonable deviations from the Missing At Random assumption. The missing data will be predicted based on the analysis of the observed data. In the first sensitivity analysis, we will reanalyze the data including the predicted value for the missing values, but add .2*SD to the missing data in the intervention group only. In the second sensitivity analysis, we will subtract .2*SD from the missing data in the intervention group only. In the third, we add .2*SD to the missing data in the TAU group only, and, in the last, subtract .2*SD from the missing data in the TAU group only.

The results for the primary outcome as a supplement will be split according to gender, single parenting (yes/no), oppositional defiant disorder/conduct disorder-comorbidity, and maternal ADHD symptoms. A number of scientific articles will be generated on the basis of collected data from this trial, including publications outlining moderator and mediator analyses. Results will be reported according to the CONSORT statement for nonpharmacological interventions [31].

**Time-Line** Funding for this trial was granted in 2011 and in 2014. Enrollment of participants started in August, 2012, and the data collection is expected to be finalized at the end of December, 2015. The primary outcome paper will be submitted for publication mid-year 2016.

## Discussion

### Attention Deficit/Hyperactivity-Disorder in Children

This trial addresses the need to investigate the effect of nonpharmacological treatments for young children with ADHD in everyday clinical practice. It is the first trial of the NFPP to be based on the recruitment of children referred directly to a child psychiatry department through official community pathways, to our knowledge. In this sense, this randomized trial tests the effectiveness of the NFPP with the children, families, and clinicians in the clinical setting for which the intervention is ultimately intended, and compares directly to the usual clinical care on offer for young children diagnosed with ADHD. The trial is also the first evaluation of the NFPP outside an English speaking context, to our knowledge.

Given the controversies surrounding the ADHD diagnoses in young children, and the challenge that confronts assessment of preschool children with ADHD symptoms, diagnostic procedures require particular attention [59]. This trial is the first to include young children with a formal clinical diagnosis of ADHD following standard clinical and DAWBA assessment, to our knowledge. The clinical assessment ensures thorough medical, psychological, and psychosocial evaluation of the child and the child’s environment, and the adjunct systematic DAWBA evaluation ensures standardization in the assessment procedure for children entering the trial.

### Reducing Chance of Bias

To reduce the chance of bias, the trial includes measures involving direct observation of children and of parenting by blinded raters as well as ratings by naïve informants (ie, day-care staff) along with parent ratings. This will allow for the investigation of rater effect on child outcome, and facilitate the exploration of whether child outcome is stable across settings.

Identifying efficacious treatments for young children with ADHD is important, but finding evidence from trials in settings created for research (eg, university labs, specialist clinics) or with participants recruited through advertisements does not guarantee that a treatment will work well in everyday clinical practice, across different populations, clinical contexts, cultures, and countries [27]. The current trial has carefully adapted the NFPP to fit clinical and cultural needs and commenced an evaluation of its effectiveness compared to the usual treatment interventions offered to young children with ADHD and their families referred through official community pathways. To these ends, the trial will provide important information about the effectiveness and replicability of the NFPP in the treatment of young children with ADHD in an everyday CAMHS setting. This level of investigation is needed to shrink the gap between intervention research and clinical practice [60].

ADHD is a disorder that presents with major personal and social costs. The immediate and long-term impact and adverse outcomes of ADHD for individuals and their families are considerable [61]. The benefits of early intervention for ADHD is an area of current scientific and public health focus, but is not fully documented [62]. It is therefore important that carefully implemented effectiveness trials of evidence-based treatments for young children are translated and implemented into different cultures with a view to enhance outcome for a broader population of children with ADHD and their families.

## Appendix

Multimedia Appendix 1 Adfærd.

Multimedia Appendix 2 Spørgeskema om barnets styrker og vanskeligheder (SDQ skema SMÅBØRN, til forælder).

Multimedia Appendix 3 Family strain.

Multimedia Appendix 4 Parenting Sense of Competence Scale, PSOC, Charlotte Johnston, University of British Columbia.

## Abbreviations

ADHD: attention deficit/hyperactivity-disorder
ADHD RS: ADHD Rating Scale-IV
ADRS-v1.1: Adult ADHD Self-Report Scale
CAMHS: child mental health services
CLB: Ms Cathy Laver-Bradbury
CONSORT: Consolidated Standards of Reporting Trials
DAWBA: Development and Well-Being Assessment
DSM-IV: Diagnostic and Statistical Manual of Mental Disorders
FSI: Family Strain Index
GHQ: General Health Questionnaire
GIPCI-R: Global Impressions of Parent-Child Interactions
MT: Dr Margaret Thompson
NFPP: New Forest Parenting Programme
PSOC: Parenting Sense of Competence Scale
SDQ: Strength and Difficulties Questionnaire
TAU: treatment as usual condition
WHO: World Health Organization
