# Supplementary material for: The Effectiveness of Parent Training as a Treatment for Preschool Attention-Deficit/Hyperactivity Disorder: Study Protocol for a Randomized Controlled, Multicenter Trial of the New Forest Parenting Program in Everyday Clinical Practice
Source: JMIR Res Protoc. 2016 Apr 13;5(2):e51. doi: 10.2196/resprot.5319 (PMC4848388; doi:10.2196/resprot.5319)
Supplement: Multimedia Appendix 1 [file resprot_v5i2e51_app1.pdf]

ADFÆRDSVURDERING - SMÅBØRNSVERSION

Barnets navn ____ Køn _______ Fødselsdato: _______________

Skema udfyldt af: _______________________________________ Dato: _______________

Relation til barnet: ☐ Mor ☐ Far ☐ Pædagog ☐ Andet ____________________

Vejledning: Sæt en cirkel om det tal, der beskriver barnets adfærd derhjemme, i dagplejen, i børnehaven eller i skolen det sidste halve år.

	Adfærd	Aldrig/ Sjældent	Nogle gange	Ofte	Meget ofte
1	Er ikke opmærksom på detaljer (skynder sig gennem aktiviteter, laver sjuskefejl)	0	1	2	3
2	Sidder uroligt eller bevæger konstant hænder og fødder	0	1	2	3
3	Har svært ved at fastholde koncentration ved opgaver eller under leg	0	1	2	3
4	Rejser sig fra sin plads, under måltider eller i andre situationer, hvor man forventes at blive siddende	0	1	2	3
5	Hører tilsyneladende ikke efter ved direkte tiltale	0	1	2	3
6	Farer for meget omkring eller klatrer i situationer, hvor det ikke er passende	0	1	2	3
7	Følger ikke instruktioner til ende og gør ikke opgaver færdig (som f.eks. "Tag din jakke og vanter og gå udenfor")	0	1	2	3
8	Har svært ved at lege stille og roligt	0	1	2	3
9	Har svært ved at organisere opgaver og aktiviteter (f.eks. vælge en aktivitet, hente de ting der skal bruges og gøre tingene i den rigtige rækkefølge)	0	1	2	3
10	Er altid I 'fuld fart' eller har 'krudt bagi'	0	1	2	3
11	Undgår opgaver, som kræver vedholdende opmærksomhed (som f.eks. at lægge puslespil, lære talrækken eller skrive sit navn)	0	1	2	3
12	Taler ustandseligt	0	1	2	3
13	Mister ting der skal bruges til opgaver eller aktiviteter (f.eks. vanter, sko, legetøj)	0	1	2	3
14	Buser ud med et svar, før spørgsmålet er afsluttet	0	1	2	3
15	Bliver let afledt	0	1	2	3
16	Har svært ved at vente på sin tur	0	1	2	3
17	Er glemsom i forbindelse med daglige aktiviteter (f.eks. glemmer legetøj eller ting der skal med hjem)	0	1	2	3
18	Afbryder eller forstyrrer andre	0	1	2	3

DO NOT COPY

McGoey, K.E.; Dupaul, Q.J; Haley, E; Shelton, T.L. (2007) Parent & Teacher ratings of ADHD in Preschool: The ADHD Ratingscale – IV Preschool Version. Oversat til dansk ved Lange, A-M; Lambek, R; Bilenberg, N (2012) ©
