# Supplementary material for: The Effectiveness of Parent Training as a Treatment for Preschool Attention-Deficit/Hyperactivity Disorder: Study Protocol for a Randomized Controlled, Multicenter Trial of the New Forest Parenting Program in Everyday Clinical Practice
Source: JMIR Res Protoc. 2016 Apr 13;5(2):e51. doi: 10.2196/resprot.5319 (PMC4848388; doi:10.2196/resprot.5319)
Supplement: Multimedia Appendix 2 [file resprot_v5i2e51_app2.pdf]

Spørgeskema om barnets styrker og vanskeligheder
(SDQ skema SMÅBØRN, til forælder)

Vi vil bede dig vurdere om beskrivelserne i skemaet Passer ikke, Passer delvist, Passer godt, på barnet. Det vil være os en stor hjælp, hvis du besvarer alle spørgsmålene, også selvom du er i tvivl eller synes, at beskrivelserne ikke helt giver mening i forhold til barnets alder. Vi vil bede dig svare ud fra barnets opførsel indenfor de sidste 6 måneder, eller indenfor dette skolekalenderår.

Barnets navn ________________________________ Dreng/Pige ________________________________

Fødselsdato ________________________________

	Passer ikke	Passer delvist	Passer godt
Tager hensyn til andre folks følelser	☐	☐	☐
Er rastløs, overaktiv, har svært ved at holde sig i ro i længere tid	☐	☐	☐
Klager ofte over hovedpine, ondt i maven eller kvalme	☐	☐	☐
Er god til at dele med andre børn (slik, legetøj, blyanter osv.)	☐	☐	☐
Har ofte raserianfald eller bliver let hidsig	☐	☐	☐
Er lidt af en enspænder, leger mest alene	☐	☐	☐
Gør for det meste, hvad der bliver sagt	☐	☐	☐
Bekymrer sig over mange ting, virker ofte bekymret	☐	☐	☐
Prøver at hjælpe, hvis nogen slår sig, er kede af det eller skidt tilpas	☐	☐	☐
Sidder konstant uroligt på stolen, har svært ved at holde arme og ben i ro	☐	☐	☐
Har mindst én god ven	☐	☐	☐
Slås ofte med andre børn eller mobber andre børn	☐	☐	☐
Er ofte ked af det, trist eller har let til gråd	☐	☐	☐
Er generelt vellidt af andre børn	☐	☐	☐
Er nem at distrahere, mister let koncentrationen	☐	☐	☐
Er utryg og klæbende i nye situationer, bliver nemt usikker på sig selv	☐	☐	☐
Er god ved mindre børn	☐	☐	☐
Siger ofte voksne imod	☐	☐	☐
Bliver mobbet eller drillet af andre børn	☐	☐	☐
Tilbyder ofte af sig selv at hjælpe andre (forældre, pædagoger, andre børn)	☐	☐	☐
Kan stoppe op og tænke sig om før han/hun handler	☐	☐	☐
Kan være ondskabsfuld overfor andre	☐	☐	☐
Kommer bedre ud af det med voksne end med andre børn	☐	☐	☐
Er bange for mange ting, er nem at skræmme	☐	☐	☐
Gør tingene færdige, er god til at koncentrere sig	☐	☐	☐

Har du andre bemærkninger eller bekymringer, kan du skrive dem her

____

____

Vend venligst skemaet - der er nogle få spørgsmål på bagsiden

DO NOT COPY

Samlet, mener du, at barnet har vanskeligheder på et eller flere af følgende områder:
Følelsesmæssigt, med koncentration, adfærd eller i samspil med andre mennesker?

NEJ	JA Mindre vanskeligheder	JA Tydelige Vanskeligheder	JA Alvorlige vanskeligheder
☐	☐	☐	☐

Hvis JA: Vær venlig at besvare følgende spørgsmål:

	Mindre end 1 måned	1-5 måneder	6-12 måneder	Mere end et år
Hvor længe har disse vanskeligheder stået på?	☐	☐	☐	☐

	Slet ikke	Kun lidt	Ret meget	Virkelig meget
Gør disse vanskeligheder barnet ulykkelig, eller ked af det?	☐	☐	☐	☐

	Slet ikke	Kun lidt	Ret meget	Virkelig meget
Påvirker disse vanskeligheder barnets dagligdag?				
Derhjemme?	☐	☐	☐	☐
I barnets forhold til jævnaldrene?	☐	☐	☐	☐
Med hensyn til indlæring?	☐	☐	☐	☐
I forbindelse med fritidsaktiviteter?	☐	☐	☐	☐

	Slet ikke	Kun lidt	Ret meget	Virkelig meget
Er disse vanskeligheder belastende for dig eller familien som helhed?	☐	☐	☐	☐

Underskrift___________________________ Dato____________________

Mor/far/anden relation (angiv venligst)____

Mange tak for din hjælp

© Robert Goodman, 2005
Dansk oversættelse ved Anne-Mette Lange*, Rikke Lambek, Charlotte Rask, Tine Houmann. Godkendt af Robert Goodman, 2012.
* Børne- og Ungdomspsykiatrisk Center, Risskov, Danmark.

DO NOT COPY

Spørgeskema om barnets styrker og vanskeligheder
(SDQ pædagogskema SMÅBØRN)

Vi vil bede dig vurdere om beskrivelserne i skemaet Passer ikke, Passer delvist, Passer godt, på barnet. Det vil være os en stor hjælp, hvis du besvarer alle spørgsmålene, også selvom du er i tvivl eller synes, at beskrivelserne ikke helt giver mening i forhold til barnets alder. Vi vil bede dig svare ud fra barnets opførsel indenfor de sidste 6 måneder, eller indenfor dette skolekalenderår.

Barnets navn ______________________________ Dreng/Pige ______________________________

Fødselsdato ______________________________

	Passer ikke	Passer delvist	Passer godt
Tager hensyn til andre folks følelser	☐	☐	☐
Er rastløs, overaktiv, har svært ved at holde sig i ro i længere tid	☐	☐	☐
Klager ofte over hovedpine, ondt i maven eller kvalme	☐	☐	☐
Er god til at dele med andre børn (slik, legetøj, blyanter osv.)	☐	☐	☐
Har ofte raserianfald eller bliver let hidsig	☐	☐	☐
Er lidt af en enspænder, leger mest alene	☐	☐	☐
Gør for det meste, hvad der bliver sagt	☐	☐	☐
Bekymrer sig over mange ting, virker ofte bekymret	☐	☐	☐
Prøver at hjælpe, hvis nogen slår sig, er kede af det eller skidt tilpas	☐	☐	☐
Sidder konstant uroligt på stolen, har svært ved at holde arme og ben i ro	☐	☐	☐
Har mindst én god ven	☐	☐	☐
Slås ofte med andre børn eller mobber andre børn	☐	☐	☐
Er ofte ked af det, trist eller har let til gråd	☐	☐	☐
Er generelt vellidt af andre børn	☐	☐	☐
Er nem at distrahere, mister let koncentrationen	☐	☐	☐
Er utryg og klæbende i nye situationer, bliver nemt usikker på sig selv	☐	☐	☐
Er god ved mindre børn	☐	☐	☐
Siger ofte voksne imod	☐	☐	☐
Bliver mobbet eller drillet af andre børn	☐	☐	☐
Tilbyder ofte af sig selv at hjælpe andre (forældre, pædagoger, andre børn)	☐	☐	☐
Kan stoppe op og tænke sig om før han/hun handler	☐	☐	☐
Kan være ondskabsfuld overfor andre	☐	☐	☐
Kommer bedre ud af det med voksne end med andre børn	☐	☐	☐
Er bange for mange ting, er nem at skræmme	☐	☐	☐
Gør tingene færdige, er god til at koncentrere sig	☐	☐	☐

Har du andre bemærkninger eller bekymringer, kan du skrive dem her

____

____

Vend venligst skemaet - der er nogle få spørgsmål på bagsiden

DO NOT COPY

Samlet, mener du, at barnet har vanskeligheder på et eller flere af følgende områder:
Følelsesmæssigt, med koncentration, adfærd eller i samspil med andre mennesker?

NEJ	JA Mindre vanskeligheder	JA Tydelige Vanskeligheder	JA Alvorlige vanskeligheder
☐	☐	☐	☐

Hvis JA: Vær venlig at besvare følgende spørgsmål:

	Mindre end 1 måned	1-5 måneder	6-12 måneder	Mere end et år
Hvor længe har disse vanskeligheder stået på?	☐	☐	☐	☐

	Slet ikke	Kun lidt	Ret meget	Virkelig meget
Gør disse vanskeligheder barnet ulykkelig, eller ked af det?	☐	☐	☐	☐

	Slet ikke	Kun lidt	Ret meget	Virkelig meget
Påvirker disse vanskeligheder barnets dagligdag?				
I barnets forhold til jævnaldrene?	☐	☐	☐	☐
Med hensyn til indlæring?	☐	☐	☐	☐

	Slet ikke	Kun lidt	Ret meget	Virkelig meget
Er disse vanskeligheder belastende for dig eller for gruppen af børn som helhed?	☐	☐	☐	☐

Underskrift______________________________ Dato________________________________

Pædagog/klasselærer/anden relation (angiv venligst)____

Mange tak for din hjælp

© Robert Goodman, 2005
Dansk oversættelse ved Anne-Mette Lange*, Rikke Lambek, Charlotte Rask, Tine Houmann. Godkendt af Robert Goodman, 2012.
* Børne- og Ungdomspsykiatrisk Center, Risskov, Danmark.

DO NOT COPY
