# Supplementary material for: The Effectiveness of Parent Training as a Treatment for Preschool Attention-Deficit/Hyperactivity Disorder: Study Protocol for a Randomized Controlled, Multicenter Trial of the New Forest Parenting Program in Everyday Clinical Practice
Source: JMIR Res Protoc. 2016 Apr 13;5(2):e51. doi: 10.2196/resprot.5319 (PMC4848388; doi:10.2196/resprot.5319)
Supplement: Multimedia Appendix 3 [file resprot_v5i2e51_app3.pdf]

Oversat til dansk med tilladelse fra professor Anne W. Riley, Johns Hopkins University, af A-M Lange & KK Frantzen, 2012, Børne- & Ungdomspsykiatrisk Regionscenter, Risskov ©

Family Strain Index

Der er fem svarmuligheder til de enkelte punkter i spørgeskemaet

0 = Aldrig
1 = Næsten aldrig
2 = Nogle gange
3 = Næsten altid
4 = Altid

Sæt kun ring om ét svar for hvert udsagn

Inden for de sidste 4 uger, hvor ofte har dit barn….

1.	Gjort, at du føler dig stresset eller bekymret.	0	1	2	3	4
2.	Begrænset din tid til afslapning eller deltagelse i sociale aktiviteter.	0	1	2	3	4
3.	Fået dig til at føle dig utilpas ved at invitere venner og familie til dit hjem.	0	1	2	3	4
4.	Været årsag til konflikter eller spændinger i familien.	0	1	2	3	4
5.	Forhindret dig i at tage visse steder sammen med dit barn (f.eks. supermarkedet/forestillinger, besøg hos venner/familie).	0	1	2	3	4
6.	Medført at du har ændret en planlagt familieaktivitet (f.eks. planer for weekenden, dagsudflugter, sociale arrangementer).	0	1	2	3	4

DO NOT COPY
