# Supplementary material for: The Effectiveness of Parent Training as a Treatment for Preschool Attention-Deficit/Hyperactivity Disorder: Study Protocol for a Randomized Controlled, Multicenter Trial of the New Forest Parenting Program in Everyday Clinical Practice
Source: JMIR Res Protoc. 2016 Apr 13;5(2):e51. doi: 10.2196/resprot.5319 (PMC4848388; doi:10.2196/resprot.5319)
Supplement: Multimedia Appendix 4 [file resprot_v5i2e51_app4.pdf]

Parenting Sense of Competence Scale, PSOC, Charlotte Johnston, University of British Columbia ©.
Oversat til dansk med tilladelse fra Charlotte Johnston 2012 af A-M Lange & KK Frantzen, Børne- & Ungdomspsykiatrisk Center, Risskov

At være forælder – Mor

Dit barns navn ____________________________________ Udfyldt den (dato) ______________________

Barnets fødselsdato _______________________________

Nedenfor står der en række udsagn. Du bedes svare på hvert enkelt udsagn og markere om du er enig eller uenig i hvert enkelt udsagn på følgende måde.

Hvis du er meget enig, sæt ring om bogstaverne **ME**
Hvis du er enig, sæt ring om bogstavet **E**
Hvis du er lidt enig, sæt ring om bogstaverne **LE**
Hvis du er lidt uenig, sæt ring om bogstaverne **LU**
Hvis du er uenig, sæt ring om bogstavet **U**
Hvis du er meget uenig, sæt ring om bogstaverne **MU**

DO NOT COPY

1.	De problemer der er ved at tage sig af et barn, er lette at løse, når man først ved, hvordan ens handlinger påvirker ens barn – en forståelse jeg har opnået.	ME	E	LE	LU	U	MU
2.	Selvom det at være forælder kunne være berigende, så er jeg frustreret nu, hvor mit barn har sin nuværende alder.	ME	E	LE	LU	U	MU
3.	Jeg går i seng om aftenen og vågner med den samme følelse – følelsen af ikke at have udrettet særlig meget.	ME	E	LE	LU	U	MU
4.	Jeg ved ikke, hvad det er, men somme tider, når jeg burde have kontrol, føler jeg mere, at jeg er den, der bliver manipuleret.	ME	E	LE	LU	U	MU
5.	Min mor var bedre forberedt på at være en god mor, end jeg er.	ME	E	LE	LU	U	MU
6.	Jeg ville være en god rollemodel at følge for en nybagt mor, så hun kan lære det nødvendige for at blive en god forælder.	ME	E	LE	LU	U	MU
7.	At være forælder er til at håndtere, og ethvert problem er nemt at løse.	ME	E	LE	LU	U	MU
8.	Et svært problem ved at være forælder er, ikke at vide om man gør det godt eller skidt.	ME	E	LE	LU	U	MU
9.	Nogle gange føler jeg, at jeg ikke får lavet noget.	ME	E	LE	LU	U	MU

10.	Jeg opfylder mine egne personlige forventninger til at have ekspertise i at tage vare på mit barn.	ME	E	LE	LU	U	MU
11.	Hvis der er nogen, der kan finde svaret på, hvad der foruroliger mit barn, er det mig.	ME	E	LE	LU	U	MU
12.	Mine talenter og interesser ligger inden for andre områder – ikke inden for det at være forælder.	ME	E	LE	LU	U	MU
13.	I betragtning af, hvor længe jeg har været mor, føler jeg mig fuldkommen fortrolig med rollen.	ME	E	LE	LU	U	MU
14.	Hvis det at være mor til et barn bare var mere interessant, ville jeg være motiveret for at gøre det bedre som forælder.	ME	E	LE	LU	U	MU
15.	Jeg tror helt ærligt, at jeg har alle de nødvendige færdigheder til at være en god mor for mit barn.	ME	E	LE	LU	U	MU
16.	At være forælder gør mig anspændt og urolig.	ME	E	LE	LU	U	MU

DO NOT COPY

Oversættelsen er foretaget ud fra internationale guidelines og den færdige danske version af PSOC er godkendt af rettighedshaver Charlotte Johnston. AM Lange og KK Frantzen har fået tilladelse til videreformidling af den danske PSOC, hvorfor alle anmodninger om anvendelse af testen skal adresseres til Børne- og Ungdomspsykiatrisk Center Risskov.

At være forælder – Far

Dit barns navn ____________________________________ Udfyldt den (dato) ______________________

Barnets fødselsdato _______________________________

Nedenfor står der en række udsagn. Du bedes svare på hvert enkelt udsagn og markere om du er enig eller uenig i hvert enkelt udsagn på følgende måde.

Hvis du er meget enig, sæt ring om bogstaverne **ME**
Hvis du er enig, sæt ring om bogstavet **E**
Hvis du er lidt enig, sæt ring om bogstaverne **LE**
Hvis du er lidt uenig, sæt ring om bogstaverne **LU**
Hvis du er uenig, sæt ring om bogstavet **U**
Hvis du er meget uenig, sæt ring om bogstaverne **MU**

DO NOT COPY

1.	De problemer der er ved at tage sig af et barn, er lette at løse, når man først ved, hvordan ens handlinger påvirker ens barn – en forståelse jeg har opnået.	ME	E	LE	LU	U	MU
2.	Selvom det at være forælder kunne være berigende, så er jeg frustreret nu, hvor mit barn har sin nuværende alder.	ME	E	LE	LU	U	MU
3.	Jeg går i seng om aftenen og vågner med den samme følelse – følelsen af ikke at have udrettet særlig meget.	ME	E	LE	LU	U	MU
4.	Jeg ved ikke, hvad det er, men somme tider, når jeg burde have kontrol, føler jeg mere, at jeg er den, der bliver manipuleret.	ME	E	LE	LU	U	MU
5.	Min far var bedre forberedt på at være en god far, end jeg er.	ME	E	LE	LU	U	MU
6.	Jeg ville være en god rollemodel at følge for en nybagt far, så han kan lære det nødvendige for at blive en god forælder.	ME	E	LE	LU	U	MU
7.	At være forælder er til at håndtere, og ethvert problem er nemt at løse.	ME	E	LE	LU	U	MU
8.	Et svært problem ved at være forælder er, ikke at vide om man gør det godt eller skidt.	ME	E	LE	LU	U	MU
9.	Nogle gange føler jeg, at jeg ikke får lavet noget.	ME	E	LE	LU	U	MU

10.	Jeg opfylder mine egne personlige forventninger til at have ekspertise i at tage vare på mit barn.	ME	E	LE	LU	U	MU
11.	Hvis der er nogen, der kan finde svaret på, hvad der foruroliger mit barn, er det mig.	ME	E	LE	LU	U	MU
12.	Mine talenter og interesser ligger inden for andre områder – ikke inden for det at være forælder.	ME	E	LE	LU	U	MU
13.	I betragtning af, hvor længe jeg har været far, føler jeg mig fuldkommen fortrolig med rollen.	ME	E	LE	LU	U	MU
14.	Hvis det at være far til et barn bare var mere interessant, ville jeg være motiveret for at gøre det bedre som forælder.	ME	E	LE	LU	U	MU
15.	Jeg tror helt ærligt, at jeg har alle de nødvendige færdigheder til at være en god far for mit barn.	ME	E	LE	LU	U	MU
16.	At være forælder gør mig anspændt og urolig.	ME	E	LE	LU	U	MU

DO NOT COPY

Oversættelsen er foretaget ud fra internationale guidelines og den færdige danske version af PSOC er godkendt af rettighedshaver Charlotte Johnston. AM Lange og KK Frantzen har fået tilladelse til videreformidling af den danske PSOC, hvorfor alle anmodninger om anvendelse af testen skal adresseres til Børne- og Ungdomspsykiatrisk Center Risskov.
